# *In vitro* intestinal digestion of lipids from the marine diatom *Porosira glacialis* compared to commercial LC n-3 PUFA products

**DOI:** 10.1371/journal.pone.0252125

**Published:** 2021-06-09

**Authors:** Lars Dalheim, Jon Brage Svenning, Ragnar Ludvig Olsen

**Affiliations:** Norwegian College of Fishery Science, UiT The Arctic University of Norway, Tromsø, Norway; Goethe-Universitat Frankfurt am Main, GERMANY

## Abstract

Marine sources of long chain omega-3 polyunsaturated fatty acids (LC n-3 PUFA) are in high demand for use in health supplements. Mass cultivated marine microalgae is a promising and sustainable source of LC n-3 PUFA, which relieves pressure on natural fish stocks. The lipid class profile from cultivated photosynthetic algae differ from the marine organisms currently used for the production of LC n-3 PUFA. The objective of this study was to compare *in vitro* intestinal digestion of oil extracted from the cold-adapted marine diatom *Porosira glacialis* with commercially available LC n-3 PUFA supplements; cod liver oil, krill oil, ethyl ester concentrate, and oil from the copepod *Calanus finmarchicus* (Calanus® oil). The changes in the free fatty acids and neutral and polar lipids during the enzymatic hydrolysis were characterized by liquid and gas chromatography. In Calanus® oil and the Ethyl ester concentrate, the free fatty acids increased very little (4.0 and 4.6%, respectively) during digestion. In comparison, free fatty acids in Krill oil and *P*. *glacialis* oil increased by 14.7 and 17.0%, respectively. Cod liver oil had the highest increase (28.2%) in free fatty acids during the digestion. Monounsaturated and saturated fatty acids were more easily released than polyunsaturated fatty acids in all five oils.

## Introduction

The market for omega-3 fatty acids in supplements, infant formulas, functional foods, and active pharmaceutical ingredients has seen substantial growth in the recent years, stimulated by scientific studies that demonstrate positive health effects of the long-chain omega-3 polyunsaturated fatty acids (LC n-3 PUFA) eicosapentaenoic acid (EPA; 20:5n-3) and docosahexaenoic acid (DHA; 22:6n-3) [1–5]. The main sources of LC n-3 PUFA for supplements are oils from fish liver, crustaceans, and small pelagic fish species that are only to a limited degree used for direct human consumption. Due to the restrictions on conventional fisheries, the annual production of fish oil is capped at approximately 1 million metric tons [6], of which about 75% is used by the aquaculture industry to make fish feed [7]. Therefore, the limited availability of fish oil and the increased demand from the aquaculture feed and nutraceutical industries has stimulated research and commercialization of alternative sources of LC n-3 PUFA.

In addition to nutraceutical oils extracted from lower trophic levels organisms in the marine food web like Antarctic krill [8] and the copepod *Calanus finmarchicus* [9], much focus has been on extracting oils from the main primary producers of LC n-3 PUFA; the marine microalgae [10,11]. Microalgae can be grown in bioreactors under photosynthetic or heterotrophic conditions, thereby avoiding the challenges of overfishing and ecological impact which may occur when harvesting organisms from natural populations. However, the lipid class composition of oils from lower trophic levels, including microalgae, often differs from traditional fish oil supplements and ethyl ester fish oil concentrates (EEC) and this may affect the human bioavailability of the fatty acids in the oils. Ultimately, the bioavailability of any oil depends on the enzymes required to hydrolyze lipids. Some enzymes function on specific substrates, such as pancreatic triacylglycerol lipase or phospholipase which hydrolyze triacylglycerol (TAG) and phospholipids, respectively. Others may target a wider range of substrates, such as pancreatic lipase related protein 2 and carboxyl ester lipase, both of which can hydrolyze TAG, cholesterol esters, phospholipids, galactolipids, and vitamin esters [12]. However, the reaction rates of these enzymes vary according to their substrates, and the bioavailability of LC n-3 PUFA therefore depend on the lipid classes they are bound in. For example, while fish oils mainly contain TAG, krill oil contains a large amount of phospholipids (PL) as well as TAG and TAG-derived molecules, i.e. diacylglycerol (DAG) and monoacylglycerol (MAG) [8] and oil from *C*. *finmarchicus* contains a very high proportion of wax esters (WE) [9]. Oils from phototrophic microalgae often contain substantial amounts of monogalactosyldiacylglycerol (MGDG) and digalactosyldiacylglycerol (DGDG) originating from photosynthetic membranes, in addition to TAG, TAG-derived lipid classes and phospholipids [13,14]. It is therefore important to measure the rate of enzymatic hydrolysis of novel oils when assessing their viability as dietary supplements.

*In vivo* studies of TAG, EEC, PL, and WE digestion, and uptake of EPA and DHA have begun to reveal the complexity of LC n-3 PUFA absorption, but due to heterogeneous study designs and mixed results it is difficult to establish conclusive bioavailabilities [15–21]. Some *in vitro* studies have been carried out to investigate the digestibility of MGDG and DGDG [22,23], whole microalga [24], TAG and EE [25], vegetable, fish and krill oils [26]. These studies have all used different methodologies for analyzing the digestibility and this makes it difficult to compare the results. The method applied when analyzing digestion should measure which fatty acids are released and/or which lipid classes are hydrolyzed, therefore methods such as the traditional pH stat method may not be the most suitable, especially when analyzing oils with complex lipid class compositions [27].

The photoautotrophic diatom *Porosira glacialis* is a large psychrophilic centric diatom with a highly desaturated fatty acid profile [28] and may therefore represent a valuable source of LC n-3 PUFA. To our knowledge, there are no previous studies in which the bioavailability of fatty acids from this or similar microalgae have been studied. In addition, *P*. *glacialis* can be cultivated using flue gases from high-emission industry, turning greenhouse gases into a resource, which in the long term may help to reduce the impact of heavy industry [29]. *P*. *glacialis* is currently cultivated in pilot scale cultivation tanks connected to a smelting plant. The objective of this study was to get a preliminary understanding of how well oil from *P*. *glacialis*, cultivated on CO_2_ containing flue gases from an industrial plant, was digested in an *in vitro* intestinal system and compare it with commercially available LC n-3 PUFA products. The changes in lipid classes in the oils during the enzymatic hydrolysis were analyzed by HPLC. Free fatty acids, polar and neutral lipids in the hydrolyzed oil samples were isolated by solid phase extraction (SPE) and the fatty acid composition determined by GC. The rate of hydrolysis was used to determine how well *P*. *glacialis* oil performed under *in vitro* digestion compared to commercial oil supplements.

## Materials and methods

### Materials

Oil from *Calanus finmarchicus* (Calanus® Oil) was commercially produced and provided by Calanus AS, Tromsø, Norway. Krill oil (k^3^, Life AS, Norway), ethyl ester concentrate (Trippel Omega-3, Biopharma, Norway) and cod liver oil (Tran Naturell, Møller’s, Norway) were bought at a local health store. Microalgae oil was extracted from *Porosira glacialis* cultivated as described later.

Dichloromethane; DCM (≥99.9%), hexane (≥99%), chloroform (99.0–99.4%), diethyl ether (≥99.8%), sulfuric acid (95–97%), porcine bile and porcine pancreatin were purchased from Sigma-Aldrich (St. Louis, MO, USA). LC-MS grade methanol; MeOH was from Fisher Scientific (Loughborough, UK). Acetic acid (≥99.8%) was purchased from Honeywell Fluka (Muskegon, MI, USA). Sodium bicarbonate, sodium acetate, isooctane (LC-grade), ethyl acetate (LC-grade), acetone (LC-grade) and isopropanol (LC-grade) were obtained from Merck (Darmstadt, Germany). Kristalon Flower® (14% N, 3.9% P) was obtained from Yara (Oslo, Norway). Sodium metasilicate pentahydrate was obtained from Permakem A/S (Lørenskog, Norway).

Lipid standards for HPLC analysis DGTS (1,2-dipalmitoyl-sn-glycero-3-O-4’-(N,N,N-trimethyl)-homoserine), SQDG (sulfoquinovosyldiacylglycerol) and phosphatidylinositol were purchased from Sigma Aldrich. Phosphatidylcholine (PC; 1,2-Dimyristoyl-sn-Glycero-3-Phosphatidylcholine), phosphatidylglycerol (PG; 1,2-Dimyristoyl-sn-Glycero-3-Phosphatidylglycerol Na Salt), phosphatidylserine (PS; 1,2-Dipalmitoyl-sn-Glycero-3-Phosphatidylserine Na salt), phosphatidylethanolamine (PE; 1,2-Dimyristoyl-sn-Glycero-3-Phosphatidylethanolamine), MGDG (hydrogenated monogalactosyldiacylglycerol), DGDG (hydrogenated digalactosyldiacylglycerol), ergosterol, triolein, diolein and monoolein were purchased from Larodan AB. Ethyl elaidate, 11-eicosenol and behenyl arachidate were purchased from Nu-Chek-Prep, Inc. Standards for the GC analysis, GLC-502 as free acids, were purchased from Nu-Chek-Prep, Inc.

### Cultivation of *Porosira glacialis*

The microalgae strain cultivated in this study was originally isolated from sediment samples collected in the Barents Sea (N 76° 27.54’, E 033° 03.54’) during a cruise in 2014. The cultivation took place outdoors in January 2019 in a 6000 L vertical column glass fiber photobioreactor, with similar cultivation conditions to those applied by Svenning, Dalheim [28]. Briefly, 100 g Kristalon Flower® and 1 L silicate stock solution (100 g sodium metasilicate pentahydrate L^-1^ fresh water) were added per 1000 L filtered seawater in the reactor. The pH was maintained below 8.0 by bubbling the tank with factory smoke containing 7–12% CO_2_. The CO_2_ in the smoke was also used as the carbon source during the cultivation. The biomass was harvested using a continuous solid-bowl centrifuge (Model PTDC, Nanjing Kingreat Machinery Company, Jiangsu, China) operated at 835 G, and the resulting biomass was collected using a spatula.

### Lipid extraction

Lipids were extracted from *P*. *glacialis* using a modification of the method of Folch, Lees [30]. Briefly, 9 grams of freeze-dried biomass were partitioned into six 50 ml FEP tubes (Thermo Scientific) and added 30 ml dichloromethane/methanol (2:1, v/v) and mixed by hand. The samples were then added 10 ml of 5% NaCl, before the organic phase from all samples were pooled and evaporated to dryness.

### *In vitro* digestion

Porcine pancreatin was used for the *in vitro* digestion of the oils basically as described by Aarak, Kirkhus [31].The individual oils were dissolved to 15 mg ml^-1^ in 0.15 M NaHCO_3_ containing 0.9% NaCl (total 8.0 ml) and added 11.8 mM porcine bile and 13.6 mg ml^-1^ of porcine pancreatin. The pH was adjusted to 7.0 with 0.1 M HCl. The samples were then incubated for 30, 60, and 180 minutes at 37°C under constant mixing using a LABINCO LD79 digital test-tube rotator (Breda, Netherland). The reaction was stopped by extracting the lipids using DCM/MeOH (2:1, v/v).

### Lipid class isolation and fatty acid analysis

Free fatty acids, polar and neutral lipids were isolated from the samples digested for 0, 30, 60 and 180 minutes by SPE using the method developed by Ruiz, Antequera [32]. The aminopropyl minicolumns (500 mg, Agilent, USA) were activated using 7.5 ml hexane before 150 μl sample in DCM/MeOH (2:1, v/v) was added. The neutral lipids were eluted with 5 ml of chloroform, the free fatty acids with 5 ml diethyl ether/acetic acid (98:2, v/v) and the polar lipids using 2.5 ml methanol/chloroform (6:1, v/v) and subsequently 2.5 ml 0.05 M sodium acetate in methanol/chloroform (6:1, v/v) and pooled. The 3 collected fractions were evaporated to dryness in pre-weighed glass vials, the weight of each lipid fraction was determined gravimetrically and then dissolved in DCM/MeOH (2:1, v/v) to 10 mg ml^-1^.

A GC-FID (6890N, Agilent Technologies) was used to quantify the fatty acids as FAMEs in the oils and in the fractions separated by SPE. The lipids were methylated using a modified version of Stoffel, Chu [33]. Briefly, 100 μl of lipid sample (10 mg ml^-1^) was added 100 μl of internal standard (C17:0 (Sigma Aldrich Co, St. Louis, MO, USA), 0.1 mg ml^-1^), 800 μl DCM and 2 ml 10% H_2_SO_4_ in MeOH and kept at 100°C for one hour and subsequently separated into lipid soluble and water soluble fractions by adding 3.5 ml hexane and 3.5 ml 5% NaCl and letting the tubes stand. The lipid soluble fractions were evaporated to dryness using nitrogen gas and dissolved in 100 μl of hexane before GC-FID analysis.

The GC conditions were as follows: Helium as carrier gas (1.6 ml min^-1^), Select FAME column (L 50 m, ID 0.25 mm and FT 0.25 μm, Agilent J&W GC Columns), the inlet temperature was 240°C (split 1:50, with a glass wool split liner) and the FID temperature was 250°C. Initially the GC oven was held at 60°C for one minute, then ramped up to 130°C (30°C min^-1^), further up to 195°C (1.3°C min^-1^) and finally up to 240°C (30°C min^-1^) for 10 minutes. To quantify the fatty acids, calibration curves were made by analyzing the ratio between individual fatty acids at concentrations 7.8125–2000 μg ml^-1^ of GLC 502 Free Acids (Nu-Chek-Prep, Elysian, MN, USA) and the internal standard, every concentration was analyzed as triplicates.

### Lipid class composition

The lipid class composition of the oils and hydrolyzed samples was investigated using an HPLC method based on Abreu, Solgadi [34]. HPLC analyses were performed on a Waters e2795 separations module, using a Supelcosil™ LC-SI 5 μm (25 cm x 4.6 mm) column (Supelco HPLC products, Bellefonte, PA, USA) set to a working temperature of 40°C and 40 μl injection volume. The lipids were quantified using a Waters 2424 ELS detector with the following settings: gain 100, nebulizer 30% heating power level, drift tube 45°C and pressure 40 PSI. The total run time was 41 minutes and the gradient profile can be seen in the supporting information file (S1 Table). Standard curves were made by analyzing 12.5–400 μg ml^-1^ of the lipid classes in triplicates. Both samples and standards were dissolved in mobile phase A/chloroform (4:1).

### Statistical analysis and data availability

All the analyses were performed in triplicates. The data are presented as means ± standard deviation. All analyses and figures were prepared using R v3.6.1. Data were considered significantly different if P<0.01 using a pairwise Tukey test. All data and the R markdown for this experiment are available from the Open Science Framework (OSF) under the name “*In vitro* digestion of lipids from the marine diatom *Porosira glacialis* compared to commercially available marine oils” (https://osf.io/rxnm9/?view_only=e4bec4deed91412aa45b1cb4833ac61a). This link also includes supplementary data containing tables of lipid classes and fatty acids, as well as heat maps of statistical tests.

## Results

### Fatty acid composition of the oils

The fatty acid composition of the oils are presented in Table 1, and show a diverse distribution with omega-3 fatty acids varying from a minimum of 29.5% in cod-liver oil (CLO) to a maximum of 73.2% in the ethyl ester concentrate (EEC). Of these, α-linolenic acid (18:3n-3) was present in minor amounts, from 0.6 to 1.5%. The main fatty acids in Calanus® Oil (CO) were 19.4% myristic acid (14:0), 15.5% stearidonic acid (SDA, 18:4n-3), 17% EPA and 12.5% DHA. CLO contained 15.6% oleic acid (18:1n-9), 14.5% eicosenoic acid (20:1 n-9), 9.1% EPA and 13.7% DHA. EEC contained 37.2% EPA and 27.5% DHA, while krill oil (KO) contained 21.6% palmitic acid (16:0), 11.0% palmitoleic acid (16:1 n-7), 11.5% oleic acid, 21.2% EPA and 10% DHA. Finally, *P*. *glacialis* oil (PGO) contained 12.7% palmitoleic acid, 35.2% hexadecatetraenoic acid (16:4 n-1), 28.9% EPA, and 4.9% DHA.

**Table 1:** Fatty acid composition (weight percent) of Calanus oil (CO), Cod liver oil (CLO), Ethyl ester concentrate (EEC), Krill oil (KO), and oil from *Porosira glacialis* (PGO) (n = 3).

	CO	CLO	EEC	KO	PGO
C14:0	19.4 ± 0.23	3.6 ± 0.03	ND	9.2 ± 0.23	4.0 ± 0.07
C14:1	0.5 ± 0.00	ND	ND	ND	ND
C16:0	9.7 ± 0.03	8.9 ± 0.02	ND	21.6 ± 0.24	3.5 ± 0.04
C16:1n-7	3.9 ± 0.04	9.6 ± 0.03	ND	11.0 ± 0.03	12.7 ± 0.01
C16:2n-4	ND	ND	ND	0.9 ± 0.03	2.3 ± 0.01
C16:3n-4	ND	ND	ND	ND	4.4 ± 0.02
C18:0	0.8 ± 0.01	2.0 ± 0.01	4.3 ± 0.03	1.1 ± 0.01	ND
C18:1n-9/C16:4n-1*	5.6 ± 0.06	15.6 ± 0.01	7.3 ± 0.04	11.5 ± 0.26	35.2 ± 0.02
C18:1n-7	ND	4.9 ± 0.04	3.0 ± 0.03	6.3 ± 0.02	ND
C18:2n-6	1.2 ± 0.02	2.6 ± 0.00	1.3 ± 0.02	1.9 ± 0.01	ND
C18:3n-3	1.5 ± 0.03	1.0 ± 0.01	1.0 ± 0.01	0.6 ± 0.01	ND
C20:0	ND	ND	1.0 ± 0.01	ND	ND
C18:4n-3	15.5 ± 0.19	4.4 ± 0.03	2.4 ± 0.03	1.7 ± 0.03	4.1 ± 0.02
C20:1n-9	3.0 ± 0.03	14.5 ± 0.01	2.4 ± 0.02	0.9 ± 0.00	ND
C20:2n-6	ND	ND	0.5 ± 0.01	ND	ND
C20:4n-6	0.8 ± 0.01	ND	2.4 ± 0.01	ND	ND
C22:1n-11	4.4 ± 0.05	7.0 ± 0.04	1.4 ± 0.01	ND	ND
C22:1n-9	1.8 ± 0.04	1.8 ± 0.02	2.2 ± 0.00	1.3 ± 0.01	ND
C20:5n-3	17.0 ± 0.24	9.1 ± 0.02	37.2 ± 0.18	21.2 ± 0.15	28.9 ± 0.10
C22:4n-6	0.5 ± 0.00	ND	ND	0.7 ± 0.01	ND
C24:1n-9	1.0 ± 0.03	ND	0.9 ± 0.01	ND	ND
C22:5n-3	0.9 ± 0.25	1.3 ± 0.02	5.2 ± 0.12	ND	ND
C22:6n-3	12.5 ± 0.59	13.7 ± 0.07	27.5 ± 0.16	10.0 ± 0.04	4.9 ± 0.07
∑ SFA	29.8 ± 0.24	14.5 ± 0.03	5.3 ± 0.04	32.0 ± 0.38	7.5 ± 0.05
∑ MUFA	20.3 ± 0.15	53.3 ± 0.07	17.3 ± 0.05	31.0 ± 0.24	12.7 ± 0.01
∑ PUFA	49.9 ± 0.39	32.1 ± 0.10	77.4 ± 0.04	37.0 ± 0.14	79.8 ± 0.06
∑ omega-3	47.4 ± 0.39	29.5 ± 0.09	73.2 ± 0.07	33.5 ± 0.13	37.8 ± 0.08

* The fatty acids C18:1n-9 and C16:4n-1 have similar retention times. Previously published GC-MS analysis on this strain showed that C16:4n-1 is one of the main fatty acids in *P*. *glacialis* oil, while C18:1n-9 was present in very low amounts [12].

ND = not detected.

### Digestibility

**Fig 1:**
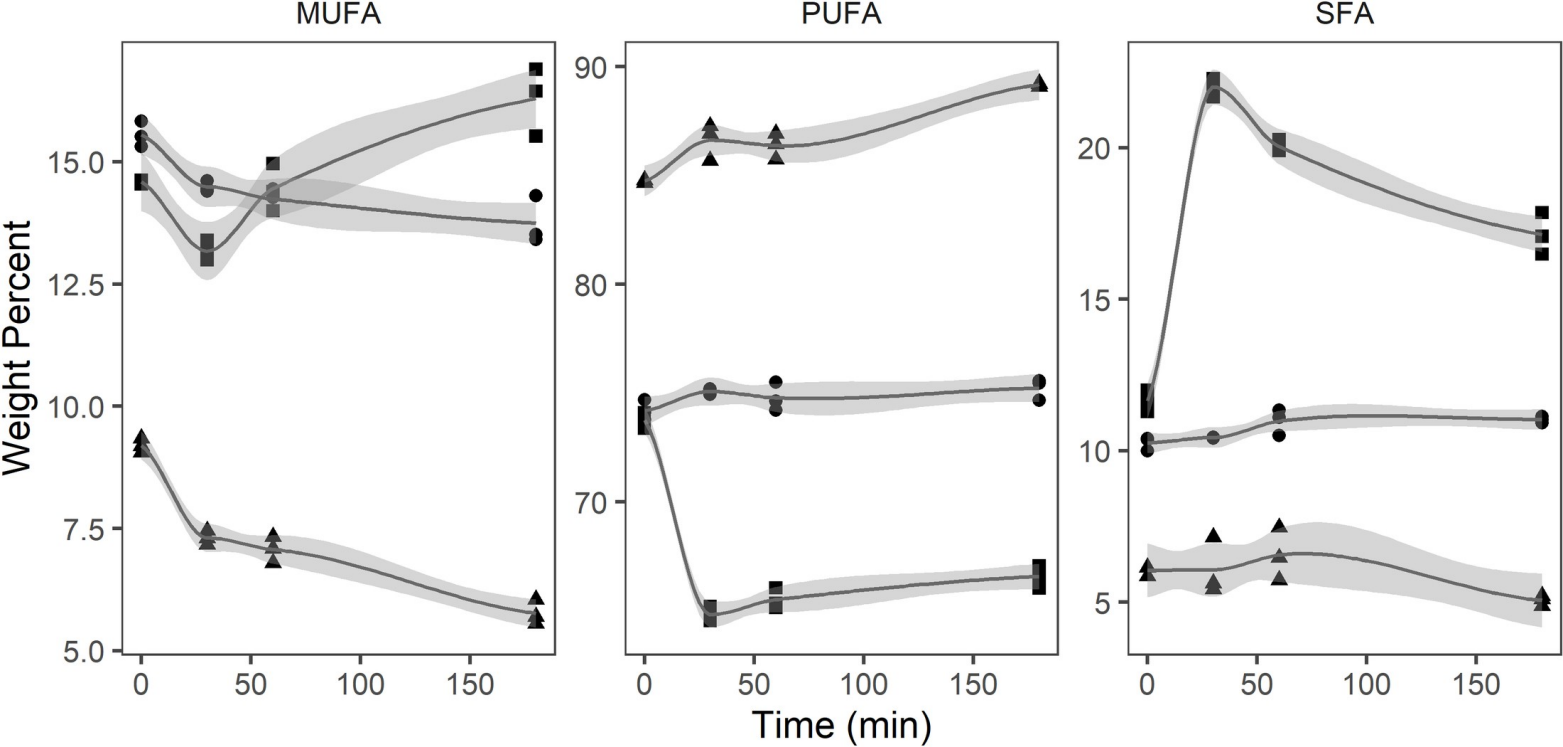
Monounsaturated fatty acids (MUFA), polyunsaturated fatty acids (PUFA), and saturated fatty acids (SFA) (weight percent) in free fatty acid (square), neutral lipid (circle) and polar lipid (triangle) fraction of oil from *Porosira glacialis* (n = 3) during *in vitro* digestion.

The oil extracted from *P*. *glacialis* displayed the most diverse lipid class composition (Table 2), differing from the other oils by containing monogalactosyldiacylglycerol (MGDG, 36.3%) and phosphatidylglycerol (PG, 23.5%). Digalactosyldiacylglycerol (DGDG, 4.1%) and free fatty acids (4.8%) were also present before digestion. PGO showed an increase in FFA from 4.8 to 12.2% between 0 and 30 minutes, and from 14.0 to 21.8% between 60 and 180 minutes. Phosphatidylglycerol (PG) and triacylglycerol (TAG) decreased from 23.5 and 4.9% to 15.3% and 1.3%, respectively, while DAG was reduced from 14.2 to 11.1%. MGDG was not affected during the initial 60 minutes of digestion, but decreased from 36.6 to 32.0% between 60 and 180 minutes. No decrease of PC was observed during digestion of PGO. The relative amount of MUFA decreased in the neutral (TAG, DAG, and MAG) and polar lipid (MGDG, DGDG, PG, and PC) fraction over time, from 15.6 to 13.7% and 9.2 to 5.8%, respectively (Fig 1). In the free fatty acid fraction, MUFA increased from 14.6 to 16.3%. PUFA remained unchanged in the neutral fraction throughout the digestion period, whereas it increased from 84.8 to 89.2% in the polar fraction and decreased from 73.8 to 66.6% in the free fatty acid fraction. SFA increased from 11.6 to 17.1% in the free fatty acid fraction, remained unchanged in the polar lipid fraction and increased from 10.3 to 11.0% in the neutral fraction.

**Table 2:** Lipid class composition (weight percent) of *Porosira glacialis* oil (time 0) and changes during in vitro digestion (n = 3).

Time (min)	0	30	60	180
TAG	4.9 ± 0.64^A^	3.4 ± 0.10^B^	3.2 ± 0.54^B^	1.3 ± 0.42^C^
DAG	14.2 ± 0.17^A^	9.7 ± 0.21^B^	11.4 ± 1.53^A^^B^	11.1 ± 2.22^A^^B^
FFA	4.8 ± 0.48^A^	12.2 ± 0.61^B^	14.0 ± 2.18^B^	21.8 ± 2.37^C^
MAG	3.8 ± 0.21^A^	2.9 ± 0.07^B^	3.1 ± 0.24^A^^B^	3.6 ± 0.47^A^^B^
MGDG	36.3 ± 0.38^A^	38.1 ± 0.42^A^	36.6 ± 1.51^A^	32.0 ± 1.62^B^
DGDG	4.1 ± 0.12^A^	5.8 ± 0.43^B^	6.2 ± 0.53^B^	5.7 ± 0.74^B^
PG	23.5 ± 0.55^A^	18.7 ± 0.13^B^	17.3 ± 0.22^B^	15.3 ± 1.09^C^
PC	8.5 ± 0.50^A^	9.2 ± 0.23^A^	8.3 ± 0.26^A^	9.4 ± 1.17^A^

^A,B,C^(Results in the same row sharing superscripted letters are not significantly different (P≥0.01)).

Triacylglycerol (TAG), diacylglycerol (DAG), free fatty acids (FFA), monoacylglycerol (MAG), monogalactosyldiacylglycerol (MGDG), digalactosyldiacylglycerol (DGDG), phosphatidylglycerol (PG), phosphatidylcholine (PC).

**Fig 2:**
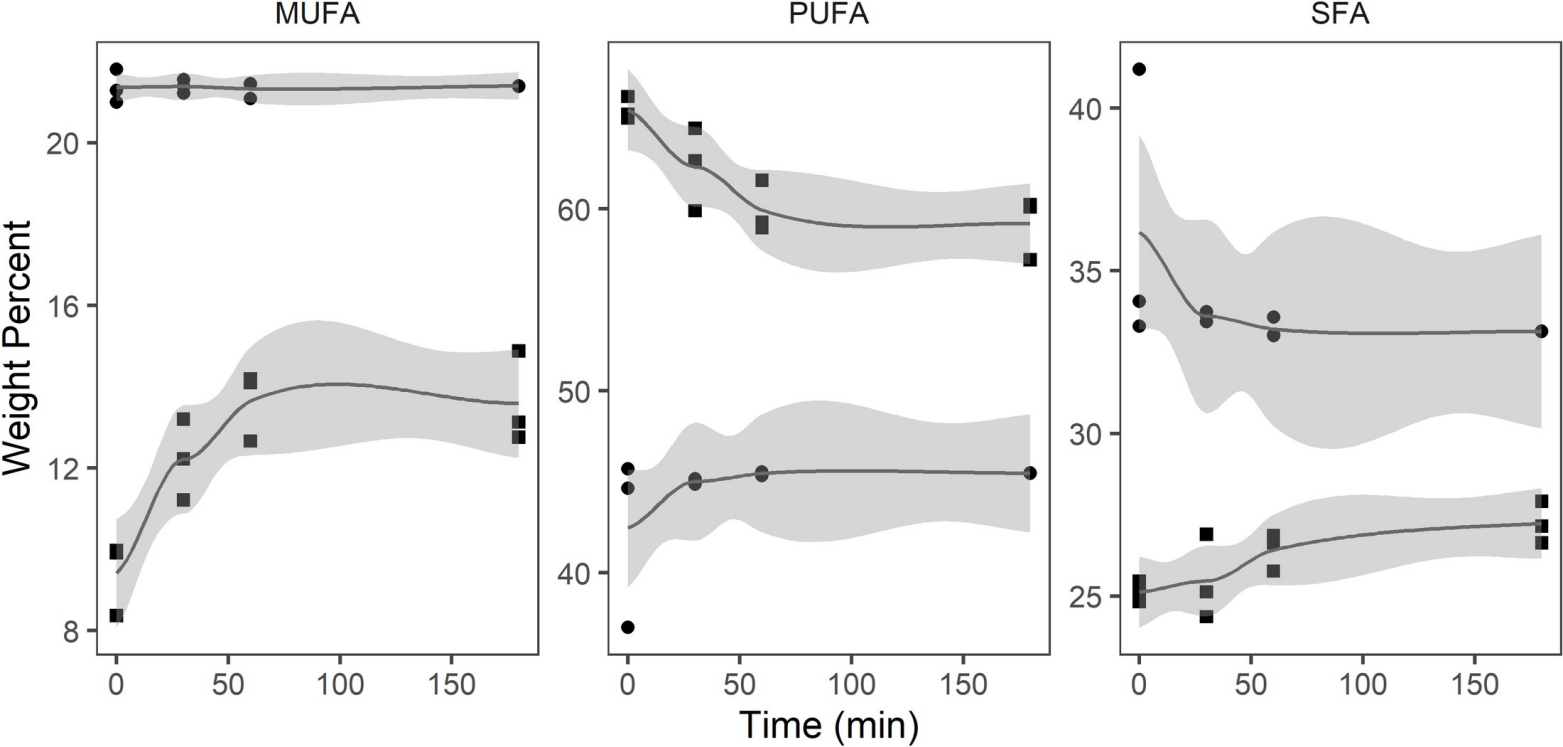
Monounsaturated fatty acids (MUFA), polyunsaturated fatty acids (PUFA), and saturated fatty acids (SFA) (weight percent) in free fatty acid (square) and neutral lipid (circle) fraction of Calanus® oil (n = 3) during *in vitro* digestion.

Prior to digestion, the dominating lipid classes in CO were wax esters (84.7%) and free fatty acids (10.2%) while fatty alcohols represented only 2.5% (Table 3). The amount of FFA in CO increased slowly but gradually from 10.2% at the beginning of the experiment to 14.2% after 180 minutes. The amount of WE appeared to decrease over time, however, the differences were not statistically significant at the threshold of p<0.01. No change in the concentration of fatty alcohols was observed. In the neutral fraction of CO (WE, TAG, DAG, and FAlc), the changes in MUFA, PUFA, and SFA were not statistically significant (Fig 2). In the free fatty acid fraction, SFA increased from 25.1 to 27.2% and PUFA decreased from 65.5 to 59.2% during the digestion. No significant changes occurred in MUFA in this fraction.

**Table 3:** Lipid class composition (weight percent) of Calanus® oil (time 0) and changes during *in vitro* digestion (n = 3).

Time (min)	0	30	60	180
WE	84.7 ± 0.76^A^^B^^C^	85.4 ± 1.44^B^	83.6 ± 1.47^A^^B^^C^	81.7 ± 1.48^C^
TAG	1.4 ± 0.15^A^	1.3 ± 0.15^A^	0.9 ± 0.60^A^	0.9 ± 0.08^A^
FAlc	2.5 ± 1.16^A^	2.0 ± 0.36^A^	2.0 ± 0.16^A^	1.9 ± 0.16^A^
DAG	1.2 ± 0.09^A^	1.2 ± 0.23^A^	1.4 ± 0.19^A^	1.4 ± 0.02^A^
FFA	10.2 ± 2.16^A^	10.1 ± 0.87^A^	12.1 ± 1.35^A^^B^	14.2 ± 1.47^B^

^A,B,C^(Results in the same row sharing superscripted letters are not significantly different (P≥0.01)).

Wax esters (WE), triacylglycerol (TAG), fatty alcohols (FAlc), diacylglycerol (DAG), free fatty acids (FFA).

**Fig 3:**
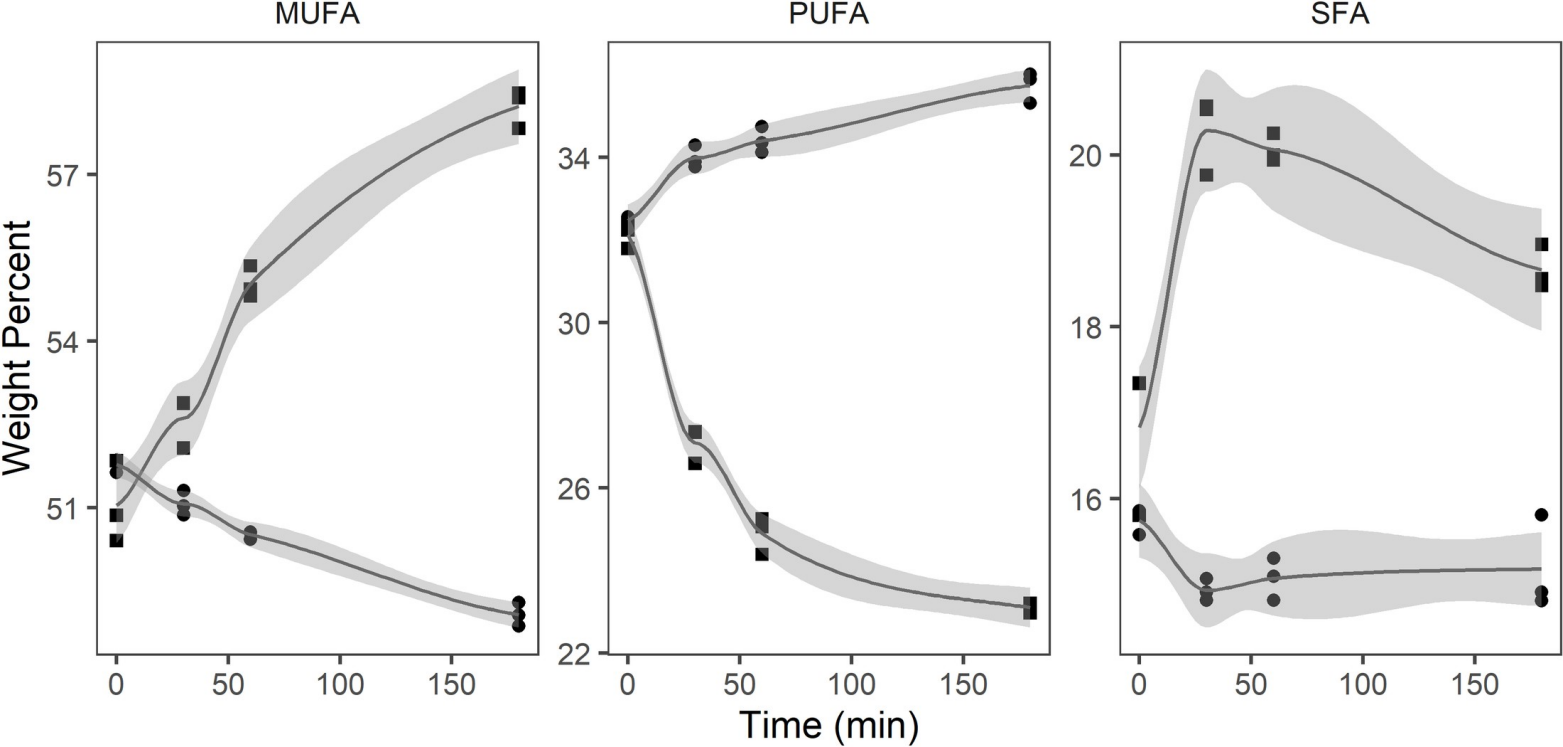
Monounsaturated fatty acids (MUFA), polyunsaturated fatty acids (PUFA), and saturated fatty acids (SFA) (weight percent) in free fatty acid (square) and neutral lipid (circle) fraction of cod-liver oil (n = 3) during *in vitro* digestion.

CLO (Table 4) initially contained 99.7% TAG, which decreased steadily to 64.4% during the 180 min *in vitro* digestion. This was accompanied by an increase of FFA, and MAG from 0 to 28.2 and 4.5%, respectively while DAG increased from 0.3 to 2.9%. In the neutral fraction of CLO (TAG, DAG, and MAG), SFA was stable at approximately 15% during the digestion (Fig 3). MUFA decreased from 51.8 to 49.1% and PUFA increased from 32.5 to 35.7% in the same fraction. In the free fatty acid fraction, SFA increased from 16.8 to 20.3% during the first 30 minutes, and then decreased to 18.7% at 180 minutes. During the digestion, PUFA decreased from 32.1 to 23.1% and MUFA increased from 51.0 to 58.2% in the free fatty acid fraction.

**Table 4:** Lipid class composition (weight percent) of cod-liver oil (time 0) and changes during *in vitro* digestion (n = 3).

Time (min)	0	30	60	180
TAG	99.7 ± 0.07^A^	90.1 ± 0.46^B^	79.1 ± 2.24^C^	64.4 ± 4.14^D^
DAG	0.3 ± 0.07^A^	0.7 ± 0.06^A^	1.8 ± 0.31^B^	2.9 ± 0.04^C^
FFA	0.0 ± 0.00^A^	8.7 ± 0.91^B^	16.0 ± 1.67^C^	28.2 ± 3.89^D^
MAG	0.0 ± 0.00^A^	0.5 ± 0.43^A^	3.1 ± 0.31^B^	4.5 ± 0.44^C^

^A,B,C,D^(Results in the same row sharing superscripted letters are not significantly different (P≥0.01)).

Triacylglycerol (TAG), diacylglycerol (DAG), free fatty acids (FFA), monoacylglycerol (MAG).

**Fig 4:**
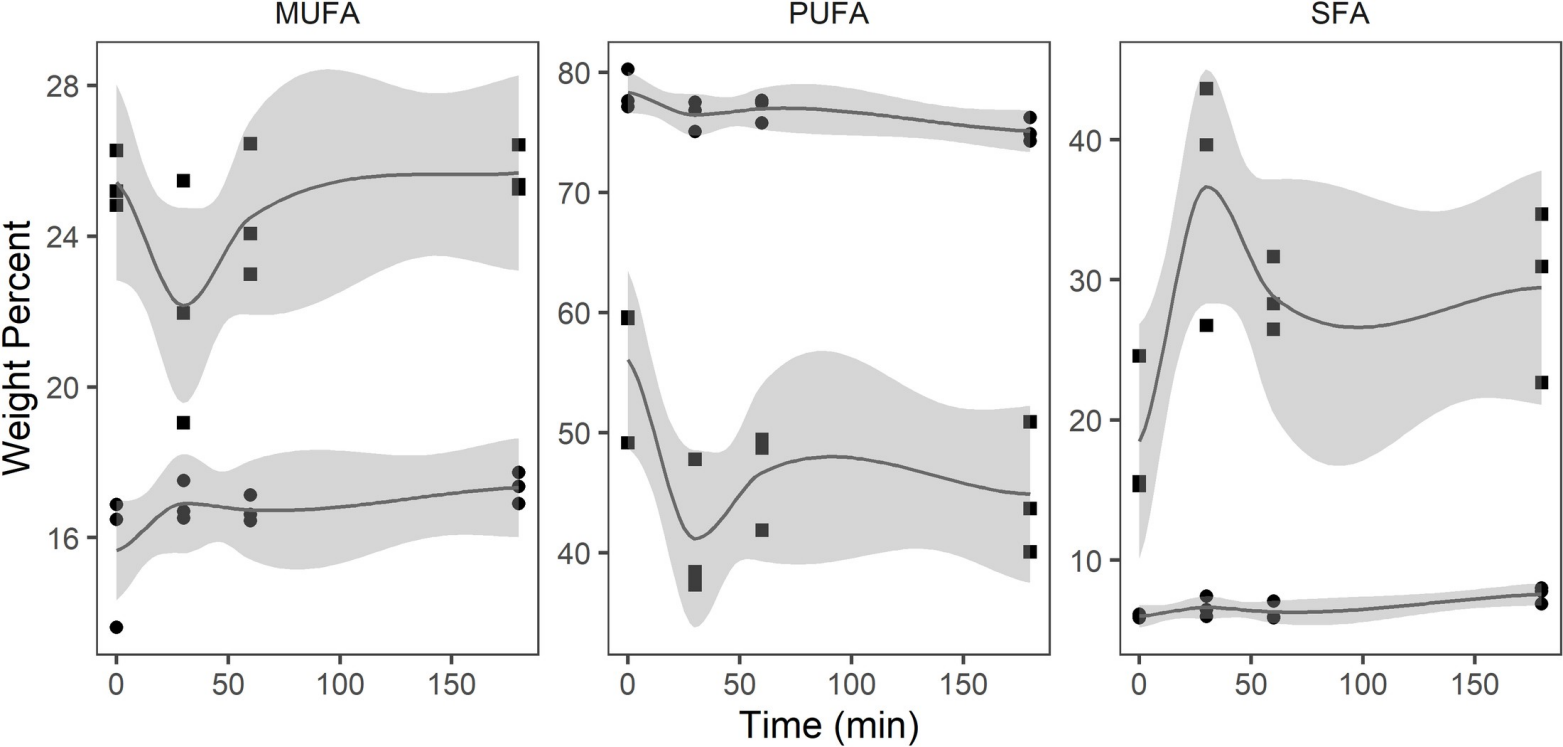
Monounsaturated fatty acids (MUFA), polyunsaturated fatty acids (PUFA), and saturated fatty acids (SFA) (weight percent) in free fatty acid (square) and neutral lipid (circle) fraction of the ethyl ester concentrate (n = 3) during *in vitro* digestion.

In EEC, fatty acid ethyl esters (EE) were the most abundant lipid class (87.3%) and a small, but not statistically significant, reduction appeared to occur during the first 30 min of the digestion (Table 5). The amount of FFA increased from 0 to 4.6% while small non-significant changes occurred in TAG and DAG during the digestion period. In the free fatty acid fraction, PUFA decreased from 56.1 to 44.9% and SFA increased from 18.5 to 29.4 during the digestion period, however, the standard deviations were large (Fig 4). In the neutral fraction (EE, TAG, and DAG), PUFA decreased from 78.3 to 75.1% and SFA increased from 6.0 to 7.6%.

**Table 5:** Lipid class composition (weight percent) of the ethyl ester concentrate (time 0) and changes during *in vitro* digestion (n = 3).

Time (min)	0	30	60	180
EE	87.3 ± 1.5^A^	83.8 ± 2.4^A^	86.9 ± 0.6^A^	84.1 ± 1.2^A^
TAG	11.9 ± 1.5^A^^B^	11.5 ± 1.6^A^	8.4 ± 0.5^B^	9.5 ± 0.6^A^^B^
DAG	0.8 ± 0.0^A^	1.4 ± 0.1^B^	1.5 ± 0.1^B^	1.8 ± 0.0^B^
FFA	0.0 ± 0.0^A^	3.3 ± 0.7^B^^C^	3.2 ± 0.2^B^	4.6 ± 0.6^B^^C^

^A,B,C^(Results in the same row sharing superscripted letters are not significantly different (P≥0.01)).

Ethyl esters (EE), triacylglycerol (TAG), diacylglycerol (DAG), free fatty acids (FFA).

**Fig 5:**
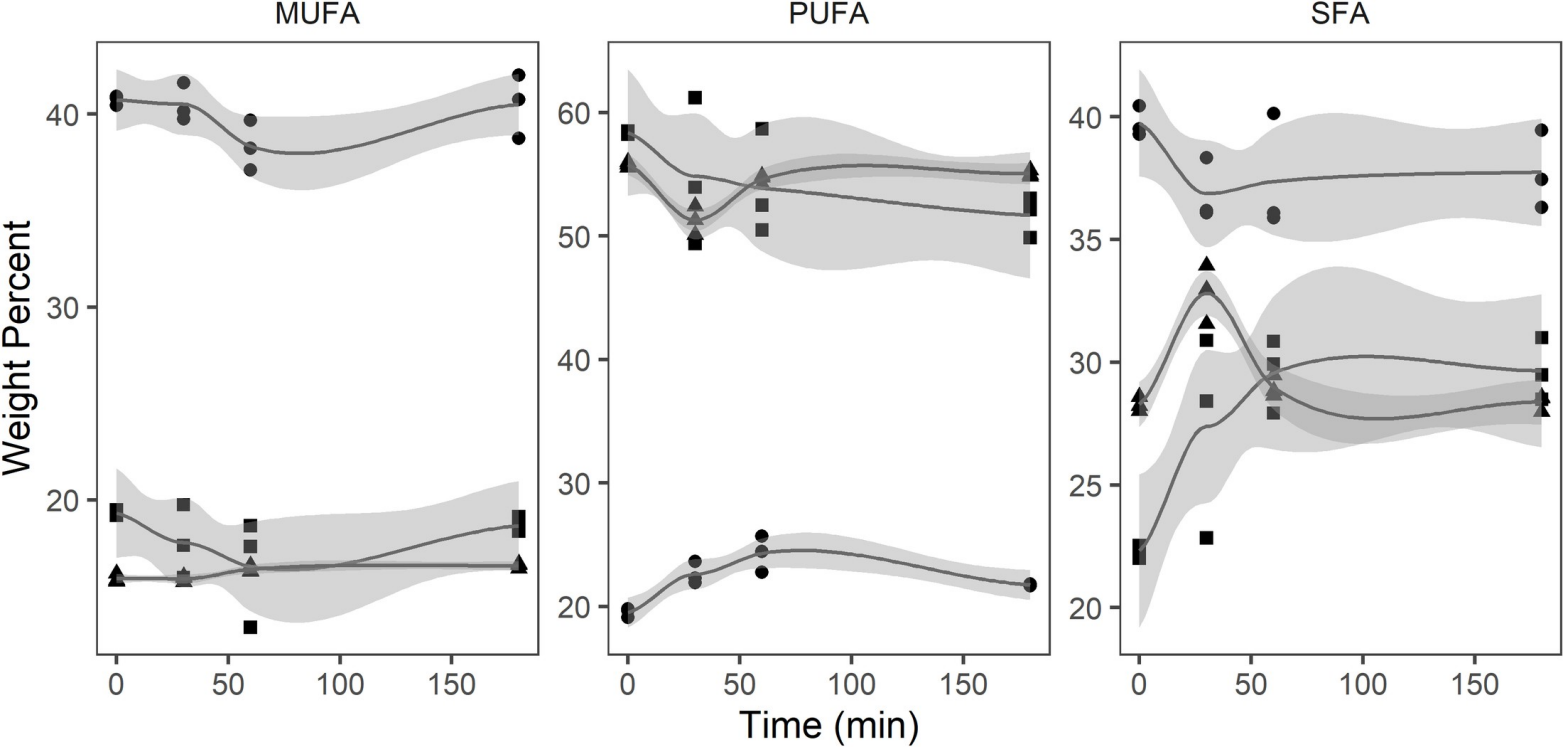
Monounsaturated fatty acids (MUFA), polyunsaturated fatty acids (PUFA), and saturated fatty acids (SFA) (weight percent) in free fatty acid (square), neutral lipid (circle) and polar lipid (triangle) fraction of krill oil (n = 3) during *in vitro* digestion.

Initially, krill oil contained primarily phosphatidylcholine (PC, 43.3%) and TAG (42.8%), however, free fatty acids (4.2%) were also present (Table 6). The FFA content increased from 4.2 to 18.9% during digestion with the fastest increase between 0 and 30 minutes. The conversion of TAG was also fastest between 0 and 30 minutes, and the total amount decreased from 42.8 to 35.4% during digestion. The amount of phosphatidylcholine (PC) decreased from 43.3 to 33.8% between 0 and 60 minutes and remained stable thereafter. During digestion, DAG decreased from 9.8 to 4.7%. MAG initially increased from 0 to 9.8% between 0 and 30 minutes, before decreasing to 6.8% at 180 minutes. In the neutral fraction (TAG, DAG, and MAG) of krill oil (Fig 5), MUFA and SFA did not show statistically significant changes with a content of approximately 40 and 38%, respectively. In the same fraction, PUFA increased from 19.5 to 21.8% during digestion. In the polar fraction (PC), the amount of MUFA, PUFA and SFA remained relatively unchanged during the digestion. In the free fatty acid fraction, PUFA decreased from 58.4 to 51.7%. Additionally, the amount of SFA increased from 22.3 to 29.7% in this fraction.

**Table 6:** Lipid class composition (weight percent) of krill oil (time 0) and changes during *in vitro* digestion (n = 3).

Time (min)	0	30	60	180
TAG	42.8 ± 1.06^A^	29.9 ± 1.99^B^	31.7 ± 4.86^B^	35.4 ± 3.05^A^^B^
DAG	9.8 ± 0.21^A^	6.0 ± 1.44^B^^C^	7.3 ± 0.20^B^	4.7 ± 0.28^C^
FFA	4.2 ± 0.07^A^	14.7 ± 3.10^B^	17.9 ± 2.25^B^	18.9 ± 0.77^B^
MAG	0.0 ± 0.00^A^	9.8 ± 2.20^B^	9.3 ± 1.47^B^	6.8 ± 0.43^B^
PC	43.3 ± 0.79^A^	39.7 ± 4.61^A^^B^	33.8 ± 1.07^B^	34.2 ± 2.11^B^

^A,B,C^(Results in the same row sharing superscripted letters are not significantly different (P≥0.01)).

Triacylglycerol (TAG), diacylglycerol (DAG), free fatty acids (FFA), monoacylglycerol (MAG), phosphatidylcholine (PC).

**Fig 6:**
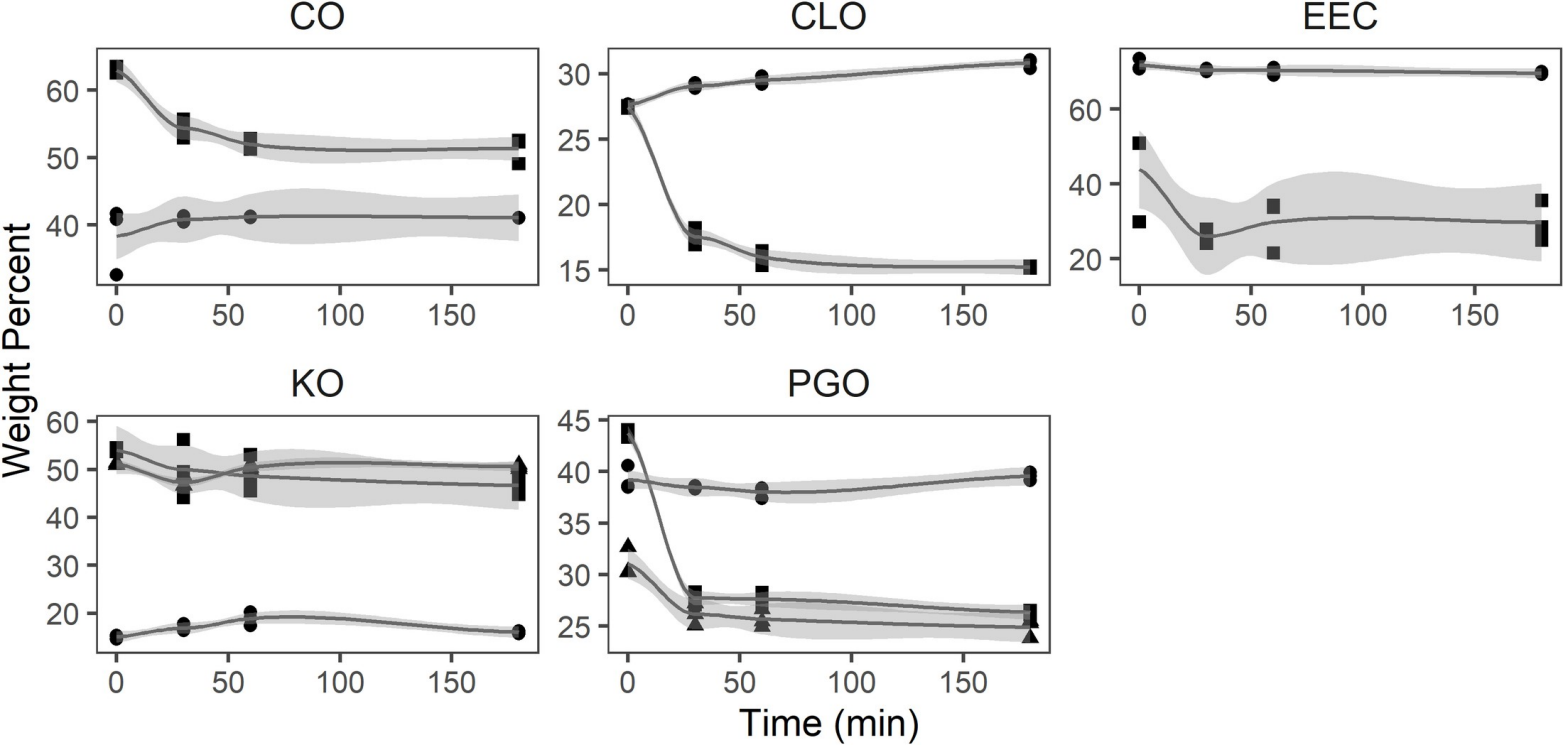
Amount of omega-3 fatty acids (weight percent) in free fatty acid (square), neutral lipid (circle) and polar lipid (triangle) fraction of samples from Calanus® oil (CO), cod-liver oil (CLO), ethyl ester concentrate (EEC), krill oil (KO) and oil from *Porosira glacialis* (PGO) (n = 3) during *in vitro* digestion.

There was a tendency towards a decrease for omega-3 fatty acids in the free fatty acid fraction of all samples: From 63.0 to 51.3% in CO, 27.5 to 15.2% in CLO, 43.9 to 29.7% in EEC, 54.0 to 46.6% in KO, and from 43.7 to 26.3% in PGO (Fig 6). The sum of omega-3 fatty acids in the neutral fraction of CO, EEC, and PGO was stable over time at approximately 41, 70, and 39%, respectively. In contrast, the relative amount of omega-3 fatty acids in the neutral fraction of CLO increased from 27.6 to 30.8% and from 15.0 to 16.0% in KO. The content of omega-3 fatty acids in the polar fraction in PGO decreased from 31.1 to 24.9%, whereas it remained unchanged in krill oil at approximately 50%.

## Discussion

The oils differed greatly in their fatty acid composition with varying amounts of SFA, MUFA and omega-3 fatty acids and were in general agreement with previously published results. However, except for the EEC, some variations in the content of the specific fatty acids will occur due to biological conditions and feeding habits of harvested organisms [8,9] and cultivating conditions of the microalgae [28].

The lipid class composition of the different oils explored in this study ranged from the simple cod-liver oil, the non-polar lipid Calanus® oil and ethyl ester concentrate to the more complex krill oil and *P*. *glacialis* oil which contained substantial amount of polar lipids in addition to the neutral lipids. The cod liver oil and the ethyl ester concentrate had no detectable free fatty acids or other polar lipids because these products have been refined during the production. The relatively high content of free fatty acids in the unrefined crustacean oils have been attributed to high lipolytic activities degrading mainly phospholipids post mortem in these species [35,36]. This explains the lack of detectable PL in the Calanus® oil. The low content of FAlc compared to FFA initially in the CO also suggests that it is primarily PL that had been degraded in *C*. *finmarchicus*. Microalgae have also been reported to have high lipolytic activity post-harvest [37] which may explain the elevated FFA level in PGO prior to digestion. The presence of FFA in nutraceutical products has been reported to be an advantage since they are independent of digestive enzymes for absorption, but the presence of other lipid classes is necessary for the free fatty acids to re-assimilate to TAG or phospholipids in the enterocyte for further transport in the lymphatic system (e.g. Lapointe, Harvey [38]). The main reason for this difference in processing of fish oil and oils from lower trophic level organisms is the low accumulation of organic pollutants in lower trophic level organisms.

As most lipid classes are too large to be absorbed into the intestinal epithelial cells, they must first be broken down into FFA and e.g. MAG, lyso-phosphlipids, lyso-galactolipids or FAlc. The *in vitro* digestion was performed using porcine pancreatin, which are extracts from the porcine pancreas that are good substitutes for the pancreatic enzymes of the human digestive system, but have slightly lower activity towards galactolipids and phospholipids than human and porcine pancreatic juices [39]. The inclusion of gastric lipase from rabbits in this study could have matched the human digestive system more closely and increased the degree of digestion [40]. However, this was a preliminary study to compare the rate of hydrolysis of oil from *P*. *glacialis* compared to commercially available omega-3 products, and since most of the lipid digestion occurs in the intestine only this part was investigated.

Cod-liver oil, oil from *P*. *glacialis*, and krill oil were more easily digested than Calanus® oil and ethyl ester concentrate in this study. The formation of FFA was highest in cod-liver oil with an increase in FFA of 28.2 percent point during the digestion period. For the *P*. *glacialis* oil and the krill oil the change in FFA were 17 and 14.7 percent point, respectively. The increase of FFA in Calanus® oil and the ethyl ester concentrate was low, indicating that the lipid classes present are hydrolyzed more slowly. Cook, Larsen [19] showed that the *in vivo* bioavailability of Calanus® oil was similar to that of ethyl ester concentrates, and previous studies have found that the *in vitro* digestion of EEC is slow [25,41]. However, Krokan, Bjerve [42], observed that even though the *in vitro* digestion of EEC was slow compared to TAG, the *in vivo* bioavailability was the same. In humans, the median transit time of food through the small intestine is approximately four hours [43], i.e. one more than the digestion period in this study. As most of the hydrolysis of EE occurred within the first thirty minutes of the digestion, adding one more hour to the *in vitro* digestion is unlikely to have affected the total digestion of EEC. Additionally, triacylglycerol was hydrolyzed more slowly in the EEC than in Krill oil and Cod-liver oil. This lower rate of hydrolysis may in part be ascribed to a matrix effect, but it is also likely that the hydrolytic enzymes have trouble hydrolyzing TAG and EE in EEC because of the high concentrations of PUFA [41]. In contrast, the digestion of Calanus® oil began between thirty and sixty minutes, suggesting that longer time is necessary for a more complete digestion of this oil. It has previously been proposed that Calanus oil is digested more slowly and that the absorption of lipids occur further down the intestinal tract [44]. However, when evaluating the results from *in vitro* digestion of lipids product inhibition will probably occur resulting in reduced release of FFA.

TAG and PC which are present in approximately equal amounts in the krill oil, were hydrolyzed at a similar rate during the *in vitro* digestion. The phospholipids PC in krill oil and PG in *P*. *glacialis* oil were both digested in this study. However, both phospholipids were present in *P*. *glacialis*, but we observed hydrolytic activity only towards PG. Roussel, Yang [45] have demonstrated that rat pancreatic lipase related protein 2 preferred PG to PC. Our results also indicate that PG is hydrolyzed more readily than PC. The galactolipids (MGDG and DGDG) which constitute about 40% of the lipid classes in the oil from *P*. *glacialis*, are also hydrolyzed by pancreatic lipase related protein 2 [46]. We found that MGDG was the only galactolipid with reduced amounts during the digestion and the onset of the hydrolysis was later than that of phospholipids and TAG. This may indicate a lower digestibility of lipids from *P*. *glacialis*. However, a recent study [39] found that porcine pancreatin had lower phospholipase and galactolipase activities than human duodenal juice. Hence, the bioavailability for humans of free fatty acids from krill oil and oil from *P*. *glacialis* may be underestimated in this study due to lower activity of the hydrolyzing enzymes. Regarding the apparent increase of DGDG during *in vitro* digestion, we cannot be sure of the cause or if the increase is real. A possible explanation may be that there is some galactosyltransferase activity in porcine pancreatin, which transfers galactose from MGDG to DGDG. This could be an interesting future study.

In krill oil and the ethyl ester concentrate the relative amount of MUFA did not increase in the free fatty acid fraction during digestion. In the other oils, MUFA did increase in this fraction. SFA, on the other hand, increased in the free fatty acid fraction of all the oils during digestion. For all the oils in this study PUFA decreased in the free fatty acid fraction during *in vitro* digestion. PUFA was more prevalent in the free fatty acid fraction prior to *in vitro* digestion than SFA and MUFA, but during digestion the release of SFA and MUFA was higher than PUFA, which explains the apparent loss of PUFA in this fraction. Our data on *in vitro* degradation of the lipids extracted from *P*. *glacialis* are in agreement with previous published results on other marine oils, which has shown that lipolysis are decreasing with increasing carbon chain length and number of double bonds of the fatty acids [47,48].

## Conclusions

In conclusion, the results showed that the lipids present in the oil extracted from the microalgae *Porosira glacialis* were hydrolyzed by porcine pancreatin at a slightly higher rate than krill oil, but at a lower rate than the TAG present in cod liver oil. The slowest hydrolysis occurred with ethyl ester concentrate and in the oil from *Calanus finmarchicus*. As with the commercial oils, PUFA were released more slowly than MUFA and SFA from the lipid classes in the oil from *P*. *glacialis*.

## Supporting information

S1 Table This is the gradient profile for the HPLC program for lipid class analysis. (DOCX)

## Abbreviations

CLO: cod-liver oil
CO: Calanus oil
DAG: diacylglycerol
DGDG: digalactosyldiacylglycerol
DHA: docosahexaenoic acid
EPA: eicosapentaenoic acid
EE: ethyl esters
EEC: ethyl ester concentrate
FAlc: fatty alcohols
FFA: free fatty acids
KO: krill oil
LC-PUFA: long-chain polyunsaturated fatty acids
MAG: monoacylglycerol
MGDG: monogalactosyldiacylglycerol
MUFA: monounsaturated fatty acid
PC: phosphatidylcholine
PG: phosphatidylglycerol
PGO: *Porosira glacialis* oil
PL: phospholipids
SFA: saturated fatty acids
SPE: solid phase extraction
TAG: triacylglycerol
WE: wax esters
