# Supplementary material for: In vitro intestinal digestion of lipids from the marine diatom Porosira glacialis compared to commercial LC n-3 PUFA products
Source: PLoS One. 2021 Jun 9;16(6):e0252125. doi: 10.1371/journal.pone.0252125 (PMC8189463; doi:10.1371/journal.pone.0252125)
Supplement: S1 Table — (DOCX) [file pone.0252125.s001.docx]

# Supporting information

Gradient profile for the HPLC program. Mobile phase A = isooctane/ethyl acetate (99.8:0.2), Mobile phase B = acetone/ethyl acetate (2:1) 0.15 % acetic acid and Mobile phase C = isopropanol/H_2_O (85:15).

| Time (min) | Mobile phase A (%) | Mobile phase B (%) | Mobile phase C  (%) | Flow (ml/min) | Curve |
| --- | --- | --- | --- | --- | --- |
| 0.0 | 100 | 0 | 0 | 1.5 | 1 |
| 1.5 | 100 | 0 | 0 | 1.5 | 6 |
| 1.6 | 97 | 3 | 0 | 1.5 | 6 |
| 6.0 | 94 | 6 | 0 | 1.5 | 6 |
| 8.0 | 50 | 50 | 0 | 1.5 | 6 |
| 8.1 | 46 | 39 | 15 | 1.5 | 6 |
| 14.0 | 43 | 30 | 27 | 1.5 | 6 |
| 14.1 | 43 | 30 | 27 | 1.5 | 6 |
| 18.0 | 40 | 0 | 60 | 1.5 | 6 |
| 23.0 | 40 | 0 | 60 | 1.5 | 6 |
| 24.0 | 0 | 100 | 0 | 1.5 | 6 |
| 25.0 | 0 | 100 | 0 | 2.0 | 6 |
| 27.0 | 0 | 100 | 0 | 2.0 | 6 |
| 27.1 | 100 | 0 | 0 | 2.0 | 6 |
| 36.0 | 100 | 0 | 0 | 2.0 | 6 |
| 36.1 | 100 | 0 | 0 | 1.5 | 6 |
